# Phytochrome A Mediates the Disassembly of Processing Bodies in Far-Red Light

**DOI:** 10.3389/fpls.2022.828529

**Published:** 2022-02-23

**Authors:** Philipp Schwenk, Andreas Hiltbrunner

**Affiliations:** ^1^Faculty of Biology, Institute of Biology II, University of Freiburg, Freiburg, Germany; ^2^Spemann Graduate School of Biology and Medicine, University of Freiburg, Freiburg, Germany; ^3^Signalling Research Centres BIOSS and CIBSS, University of Freiburg, Freiburg, Germany

**Keywords:** phytochrome, p-bodies, photomorphogenesis, light signalling, liquid-liquid-phase-separation

## Abstract

Phytochromes are red- and far-red light receptors that control the growth and development of plants, enabling them to respond adequately to changing light conditions. It has been shown that halted mRNAs stored in RNA granules called processing bodies are released upon light perception and contribute to the adaptation to the light environment. However, the photophysiological background of this process is largely unknown. We found that light of different wavelengths can trigger the disassembly of processing bodies in a dose- and time-dependent manner. We show that phytochromes control this process in red- and far-red light and that cytoplasmic phytochrome A is sufficient and necessary for the far-red light-induced disassembly of processing bodies. This adds a novel, unexpected cytoplasmic function to the processes controlled by phytochrome A. Overall, our findings suggest a role of phytochromes in the control of translationally halted mRNAs that are stored in processing bodies. We expect our findings to facilitate understanding of how light and environmental cues control the assembly and disassembly of processing bodies, which could have broader implications for the regulation of non-membranous organelles in general.

## Introduction

Throughout their life cycle, plants are subject to constantly changing environmental conditions at the place where they germinated. In order to cope with fluctuations in the surrounding, plants sense and integrate environmental cues and react with a high level of plasticity to adapt their growth and development accordingly (Chevin and Lande, 2015). This integration happens at different levels, including gene expression (Peschke and Kretsch, 2011), translation, and degradation of proteins (Wu et al., 2019), as well as modifications of mechanical properties of cell walls (Falcioni et al., 2020). Environmental cues can change rapidly, therefore reaction time is a critical factor. A mechanism to overcome the need for mRNA synthesis prior to protein translation is the storage of mRNAs in translationally inactive state (Merchante et al., 2017). The formation and disassembly of processing bodies (p-bodies), a sub-class of RNA granules, is a mechanism to store translationally halted mRNAs and release them in response to specific conditions that has recently gained attention in different model organisms and contexts (Decker and Parker, 2012; Wang et al., 2018; Jang et al., 2019).

RNA granules are cytoplasmic non-membranous organelles formed by phase separation (Sachdev et al., 2019). They can be roughly subdivided into stress granules, p-bodies, and neuronal granules (Anderson and Kedersha, 2006). To our knowledge, the first morphological description of RNA granules dates back to 1865 (Mecznikoff, 1865), though their composition and function remained unknown. Later, yeast p-bodies have been characterised and shown to consist mainly of RNA and proteins supposedly involved in degradation of mRNA (Sheth and Parker, 2003; Parker and Sheth, 2007). Experiments also suggested that primarily mRNA degrading proteins are present in plant p-bodies, indicating a conserved role throughout the phylum of eukaryotes (Maldonado-Bonilla, 2014).

Pioneering work from Hubstenberger et al. (2017) was of particular importance for understanding the protein composition of p-bodies. Increasing evidence suggests that the primary role of p-bodies is the storage of halted mRNAs. P-bodies can be disassembled upon diverse stimuli, releasing these halted mRNAs, and enabling the translation of the transcripts initially stored in p-bodies (Brengues et al., 2005; Aizer et al., 2014; Hubstenberger et al., 2017). This provides a shortcut for rapid changes of the proteome in response to environmental cues, avoiding the need for *de novo* transcribing, splicing, exporting, and maturating mRNA. In plants, this stimulus-induced disassembly plays a role in developmental processes and adaptions to the environment. It has been shown that the light conditions to which plants are exposed determine the number and size of p-bodies and thus the release of mRNAs for translation, including *GUN5* and *OE33* (Jang et al., 2019). The proteins encoded by these mRNAs are relevant for the establishment of fully developed chloroplasts and help plants to adapt to light.

Light is one of the most important factors determining a plant’s life (Paik and Huq, 2019). In order to acquire information about the light conditions in the ambient environment, plants have evolved a set of photoreceptors that monitor the light spectrum from UV-B to far-red (FR) light. Phytochromes mediate light signalling in the red (R) and FR light range of the light spectrum (Legris et al., 2019). They are synthesised in the inactive Pr form and can undergo conversion to the active Pfr form upon light absorption. Pfr can revert to Pr either upon light absorption or via thermal relaxation (Klose et al., 2015a). This behaviour forms a molecular toggle switch allowing phytochromes to determine the R:FR ratio in the surrounding environment. PhyB plays a dominant role in R light, whereas phyA is the only photoreceptor in *Arabidopsis thaliana* that mediates responses to FR light. Photoactivated phytochromes translocate to the nucleus where they interact with a plethora of signalling components to control gene expression (Legris et al., 2019). A canonical, well-investigated pathway of phytochrome signalling depends on the PHYTOCHROME INTERACTING FACTORs (PIFs), a set of bHLH transcription factors. Upon binding of PIFs to phytochromes in the nucleus, the DNA-binding activity of PIFs is suppressed (Legris et al., 2019). Another key component in phytochrome signalling is the CONSTITUTIVELY PHOTOMORPHOGENIC 1/SUPPRESSOR OF PHYA-105 (COP1/SPA) complex, which targets positive factors of photomorphogenesis, such as ELONGATED HYPOCOTYL 5 (HY5), for degradation in darkness. This complex is reorganised and inactivated upon binding of photoactivated phytochromes, allowing positive factors of photomorphogenesis to accumulate (Sheerin and Hiltbrunner, 2017). Jang et al. (2019) showed that the *hy2* mutant is unable to react to white light with a reduction of p-body numbers. This mutant is deficient in the synthesis of phytochromobilin and therefore lacks photoactive phytochromes (Kohchi et al., 2001). Additionally, it was shown that a mutation in *COP1*, *cop1-6*, leads to decreased p-body numbers in darkness. This is in line with the light-independent activation of light signalling in the *cop1-6* mutant (Ma et al., 2002). These results indicate an involvement of the phytochrome signalling system in the disassembly of p-bodies in response to light. Yet, the wavelength- and fluence rate-dependency of p-body disassembly has not been investigated and it is still unknown which specific phytochromes are involved in this process.

In our recent work, we described NOT9B as a p-body localised protein. NOT9B is part of the CCR4-NOT complex, a multi-protein complex described to be localised in p-bodies and the nucleus (Maldonado-Bonilla, 2014; Collart, 2016). This complex fulfils a multitude of different functions in plants and has gained attention in plant science over the last years. Only recently, the complex as such was shown to exist in plants (Arae et al., 2019; Zhou et al., 2020; Schwenk et al., 2021). Components of the complex have been described as regulators of transcription and integrators of environmental stresses (Liang et al., 2009; Walley et al., 2010; Suzuki et al., 2015). Here, we used YFP-tagged NOT9B as marker for p-bodies to further investigate the role of light signalling in the disassembly of p-bodies. We determined the basic physiological parameters that control this process, such as the light intensity and quality. P-body disassembly in different mutant and transgenic backgrounds was analysed to evaluate photoreceptor dependency of this process.

## Results

### Light of Different Wavelengths Triggers p-Body Disassembly

**FIGURE 1:**
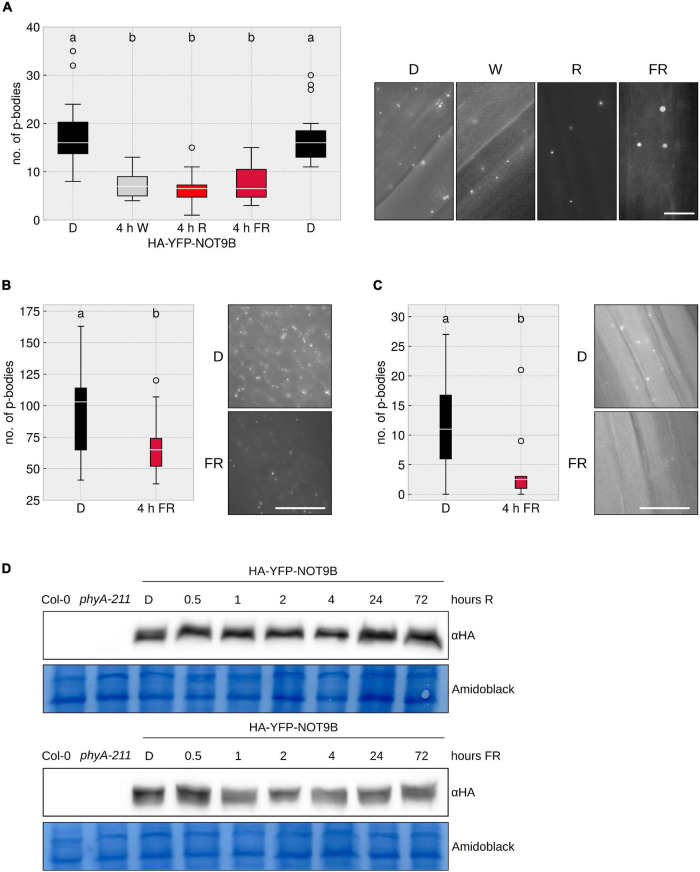
Different wavelengths trigger p-body disassembly. **(A–C)** HA-YFP-NOT9B tagged p-bodies in different light conditions in different tissues. *p35S:HA-YFP-NOT9B* expressing seedlings were grown for 4 days in darkness, followed by 4 h of indicated light quality at a fluence rate of 40 μmol m^–2^s^–1^ (FR) or 20 μmol m^–2^s^–1^ (R). Seedlings were imaged and 15 pictures were evaluated. One representative experiment out of three independent experiments is shown. Representative microscope pictures for each treatment are displayed. Different letters indicate significant differences between groups as determined by one-way ANOVA, followed by Tukey’s HSD. *P* < 0.05. Scale bar represents 10 μm. Number of p-bodies was quantified in **(A)** hypocotyl epidermal cells, **(B)** cotyledon mesophyll cells, and **(C)** root epidermal cells. **(D)** Protein levels of HA-YFP-NOT9B remain constant under light treatments. 4-day old, dark-grown HA-YFP-NOT9B expressing seedlings were treated with light (40 μmol m^–2^s^–1^ FR or 20 μmol m^–2^s^–1^ R) for the indicated time. Total protein was extracted and analysed by Western blotting; HA-YFP-NOT9B was detected using αHA antibody; the Amido Black stained membrane is shown as loading control.

As demonstrated by Jang et al. (2019), prolonged exposure to white light triggers a reduction of the number of p-bodies in cotyledons. To further investigate this effect, we performed similar experiments using *Arabidopsis thaliana* lines stably expressing the p-body localised protein NOT9B N-terminally fused to HA-YFP. We found that 4 h of exposure to FR or R light reduced p-body numbers in hypocotyl epidermis cells (Figure 1A). This effect is tissue independent, as we also observed an FR light-dependent reduction of p-body numbers in roots and cotyledons of the HA-YFP-NOT9B transgenic line (Figures 1B,C). The effect of FR light on p-bodies is possibly independent of photosynthesis and the energy status, since FR light is poorly photosynthetically active. Supporting this notion, we found that supplementing the growth medium with sucrose does not have any effect on the number of p-bodies (Supplementary Figure 1). To investigate the possibility that light has an effect on the protein level of NOT9B, we analysed the stability of HA-YFP-NOT9B in R and FR light by western blotting. The amount of NOT9B is not or only slightly affected by light treatments that trigger p-body disassembly. Differences in HA-YFP-NOT9B levels did not exceed 30% and there was no trend toward lower levels after longer exposure to light (Supplementary Figure 2). Therefore, the lower abundance of p-bodies in light cannot be explained by reduced levels of HA-YFP-NOT9B (Figure 1D).

Using another p-body marker, DCP1-CFP, we also observed a reduction in p-body numbers in response to FR light, similar to HA-YFP-NOT9B marked p-bodies. This indicates that light does not specifically trigger the exclusion of HA-YFP-NOT9B from p-bodies but rather promotes a general disassembly of p-bodies (Supplementary Figure 3).

### P-Body Disassembly in Far-Red and Red Light Is Dependent on Phytochrome A and B, Respectively

**FIGURE 2:**
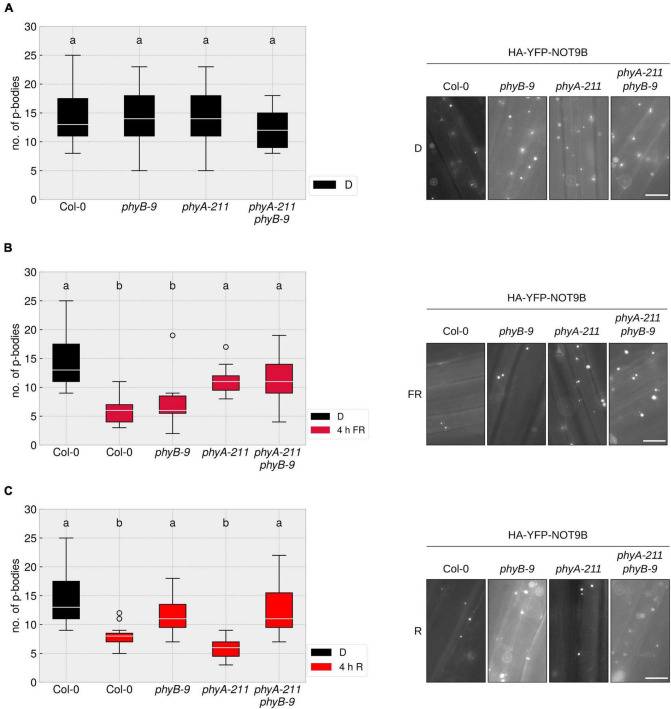
R and FR light-induced reduction of p-body abundance is dependent on phytochromes. **(A–C)** *p35S:HA-YFP-NOT9B* expressing seedlings in the indicated genetic backgrounds were grown for 4 days in darkness. Abundance of p-bodies was quantified before exposure to light **(A)**, or following 4 h exposure to FR **(B)** or R **(C)** light. Light intensity was set to a fluence rate of 40 μmol m^–2^s^–1^ (FR) or 20 μmol m^–2^s^–1^ (R). Epidermis cells of hypocotyls of seedlings were imaged and 15 pictures were evaluated. One representative experiment out of three independent experiments is shown. Representative microscope pictures for each treatment are displayed. Different letters indicate significant differences between groups as determined by one-way ANOVA, followed by Tukey’s HSD. *P* < 0.05. Scale bar represents 10 μm.

So far, it was unclear which photoreceptor mediates the disassembly of p-bodies in response to different light qualities. We crossed the HA-YFP-NOT9B expressing line into *phyA-211*, *phyB-9*, and *phyA-211 phyB-9* mutants and quantified p-body number reduction in response to R and FR light. The reduction of p-body numbers in response to R light was dependent on phyB, whereas the reduction in FR light required phyA. The *phyA-211 phyB-9* double mutant was not able to react to any of these light qualities in terms of reduction of p-body numbers (Figures 2A–C). This behaviour is in line with the canonical function of phyA and phyB as primary FR and R light receptors, respectively.

NOT9B directly interacts with phyA, raising the possibility that the phyA-induced disassembly of p-bodies depends on this interaction. Therefore, we took advantage of a point mutant of NOT9B, NOT9B ΔPNB, which lacks phyA binding capability, to examine the effect of direct physical interaction of phyA with the p-body marker NOT9B (Schwenk et al., 2021). As shown in Supplementary Figure 4, the number of p-bodies in FR light was reduced to a similar degree in lines expressing HA-YFP-NOT9B ΔPNB or HA-YFP-NOT9B, indicating that the disassembly of p-bodies in FR light is independent of NOT9B’s direct interaction with phyA, even though it is dependent on the presence of phyA.

### P-Body Disassembly in Response to Light Is Dependent on the Time and Intensity of Illumination

**FIGURE 3:**
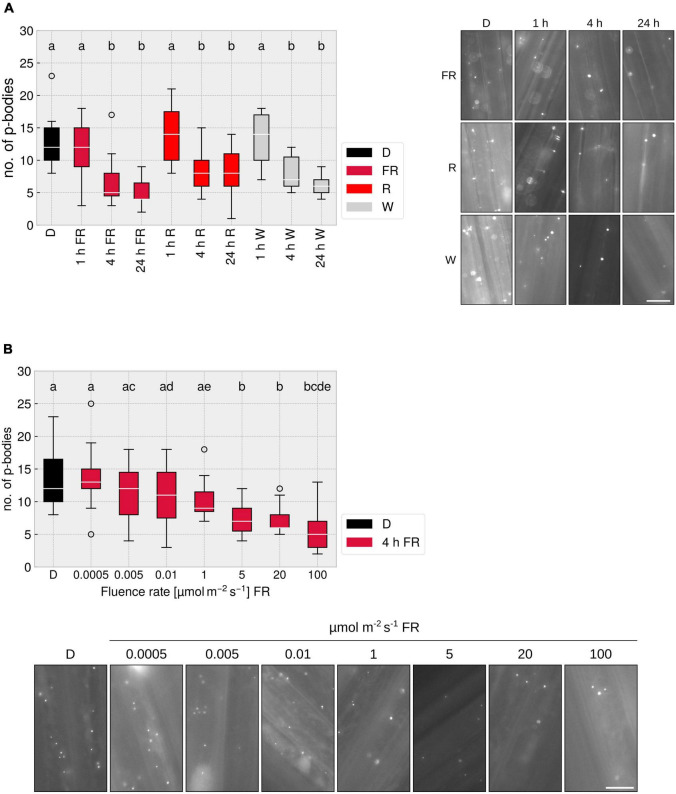
FR light-dependent reduction of p-body abundance is time- and fluence rate dependent. **(A)** p-body disassembly kinetics. 4-day old, dark-grown *p35S:HA-YFP-NOT9B* expressing seedlings were grown under indicated light conditions for different time spans. Light intensity was set to a fluence rate of 40 μmol m^–2^ s^–1^ (FR) or 20 μmol m^–2^ s^–1^ (R). Epidermis cells of hypocotyls of seedlings were imaged and 15 pictures were evaluated. One representative experiment out of three independent experiments is shown. Representative micrograph pictures for each treatment are displayed. Different letters indicate significant differences between groups as determined by one-way ANOVA, followed by Tukey’s HSD. *P* < 0.05. Scale bar represents 10 μm. **(B)** Fluence rate dependency of p-body disassembly. 4-day old, dark-grown *p35S:HA-YFP-NOT9B* expressing seedlings were exposed to FR light of different fluence rates for 4 h. Epidermis cells in hypocotyls of seedlings were imaged and 15 pictures were evaluated. One representative experiment out of three independent experiments is shown. Representative microscope pictures for each treatment are displayed. Different letters indicate significant differences between groups as determined by one-way ANOVA, followed by Tukey’s HSD. *P* < 0.05. Scale bar represents 10 μm.

Many responses mediated by phytochromes, e.g., the inhibition of hypocotyl elongation, are dependent on the fluence rate and the light quality (Casal et al., 2014). To investigate these characteristics in the context of p-body disassembly, we treated plants for different time-spans with R, FR, or W light. Illumination for 1 h did not lead to a significant reduction in p-body number, yet 4 h exposure to either R, FR, or W light triggered an approx. 50% reduction in p-body abundance; exposing the seedlings for 24 h to light did not further reduce the number of p-bodies (Figure 3A). A reaction after 4 h is known for some phytochrome mediated responses, e.g., upregulation of gene expression (*ELIP1/2*, *CHS*) (Peschke and Kretsch, 2011) or the unfolding of cotyledons (Kretsch, 2010). To investigate whether the reduction of p-body disassembly is dependent on the intensity of FR light used for illumination, we treated the plants for 4 h with FR light of different intensities. A clear dose-dependency was observed, with a saturation point between 5 and 20 μmol m^–2^s^–1^ (Figure 3B). This is similar to canonical responses such as inhibition of hypocotyl growth in FR light. The non-saturating effect of lower intensities might be due to a reduced total fluence. To assay this, we increased illumination to 24 h, yielding very similar results as 4 h (Supplementary Figure 5). This indicates that the fluence rate rather than the total fluence is determining the degree of p-body disassembly in FR light, suggesting that this response is a high-irradiance response (HIR) mediated by phyA.

### Cytoplasmic phyA Is Sufficient to Reduce the Number of Processing Bodies

To date, by far the most phyA-mediated responses that have been investigated are dependent on nuclear localised, light-activated phyA, whereas only very few responses, such as the control of translation of *PORA* mRNA, are described to be mediated by cytoplasmic phyA (Paik et al., 2012; Hughes, 2013). Since p-bodies are localised to the cytoplasm, the question arises whether the disassembly of p-bodies is mediated by a cytoplasmic signalling pathway or routed through the nucleus, e.g., via transcription of a factor that is active in the cytoplasm. PhyA nuclear import upon light perception depends on FHY1 and FHL (Hiltbrunner et al., 2006), making the *fhy1-3 fhl-1* mutant an ideal tool to tackle this question.

**FIGURE 4:**
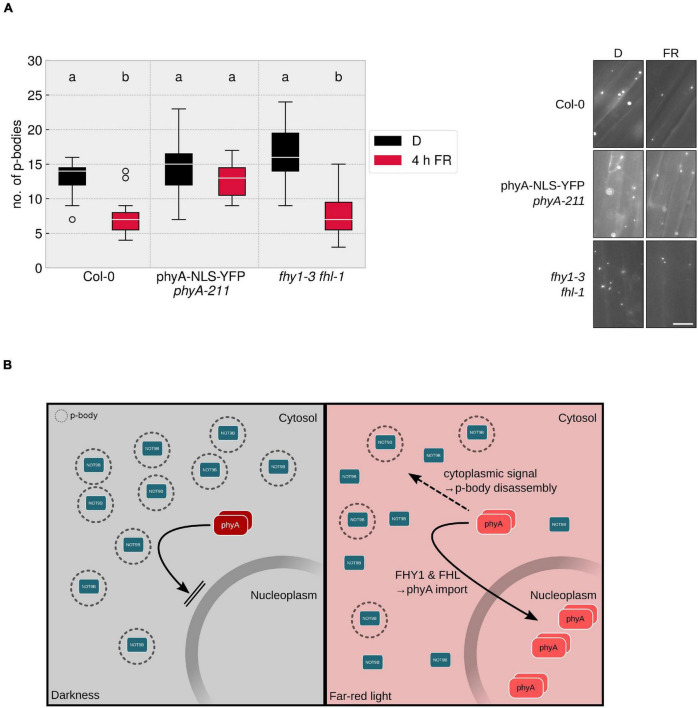
Cytoplasmic phyA is sufficient and necessary for FR light-dependent reduction of p-body numbers. **(A)** *p35S:HA-YFP-NOT9B* expressing plant lines were crossed into *phyA-211 pPHYA:PHYA-NLS-YFP* and *fhy1-3 fhl-1*. 4-day old, dark-grown seedlings were treated for 4 h with FR light (40 μmol m^–2^s^–1^) and analysed. Epidermis cells in hypocotyls of seedlings were imaged and 15 pictures were evaluated, focusing on the cytoplasmic p-body fraction marked by HA-YFP-NOT9B. One representative experiment out of three independent experiments is shown. Representative microscope pictures for each treatment are displayed. Different letters indicate significant differences between groups as determined by one-way ANOVA, followed by Tukey’s HSD. *P* < 0.05. Scale bar represents 10 μm. **(B)** Hypothetical model of phyA mediated p-body disassembly. In darkness, phyA is localised to the cytoplasm in its inactive Pr form; FHY1 and FHL transport part of the active phyA into the nucleus, while the remaining, cytoplasmic Pfr phyA is triggering p-body disassembly via a cytoplasmic signalling cascade.

A line expressing HA-YFP-NOT9B in the *fhy1-3 fhl-1* mutant background was evaluated for FR dependent reduction of p-body abundance. Lack of nuclear transport of phyA had no significant effect on the total number of p-bodies and the reduction in response to a 4 h FR light treatment was in the range of the line expressing HA-YFP-NOT9B in WT background (Figure 4A). This indicated that cytoplasmic phyA is sufficient to induce p-body disassembly. To test if cytoplasmic phyA is required for this response, we investigated p-body disassembly in a double transgenic Arabidopsis line co-expressing *p35S:HA-YFP-NOT9B* and *pPHYA:PHYA-NLS-YFP* in the *phyA-211* background. We could not observe any reduction of p-body numbers in response to exposure to FR light in this line containing exclusively nuclear localised phyA (Rausenberger et al., 2011). This indicated that the nuclear fraction of phyA does not play a role in this process in FR light (Figure 4A). In order to confirm that a C-terminally tagged version of phyA is generally capable of mediating the disassembly of p-bodies, we crossed *p35S:HA-YFP-NOT9B* into *phyA-211 pPHYA:PHYA-CFP*. In this line, the reduction of p-body numbers in response to light was similar to the Col-0 background (Supplementary Figure 6).

The dependency of p-body disassembly on phytochromes raises the possible involvement of canonical phytochrome downstream signalling pathways. One of the key factors that mediate signalling events downstream of phyA and phyB is HY5. Therefore, we crossed the line expressing HA-YFP-NOT9B into the *hy5-215* mutant and evaluated the response to FR light. As shown in Supplementary Figure 7, the lack of functional HY5 has no effect on the reduction of p-body numbers in response to FR light. Taken together, we conclude that the effect of phyA on p-bodies is exclusively mediated by the cytoplasmic fraction of phyA (Figure 4B). Knock-out of one of the most prominent transcription factors in light signalling, HY5, did not affect the p-body disassembly. This would be in line with the notion that there is no need for an indirect signalling via *de novo* transcription in the nucleus. In conclusion, cytoplasmic phyA might exert control over p-body number directly.

## Discussion

Light signalling mediated by phytochromes has been researched for more than 70 years, and still our knowledge of these processes is incomplete. In their pioneering work, Jang et al. (2019) showed that mRNAs crucial for kickstarting photosynthesis are halted and stored in DCP2-marked p-bodies and released upon light irradiation, thereby initiating translation of these mRNAs. So far, it was only shown that the marker protein DCP2 is excluded from p-bodies in response to light. Here, we provide evidence that other p-body markers (DCP1 and NOT9B) display a similar behaviour as DCP2. This supports the notion that the p-bodies indeed disassemble, rather than that specific factors are selectively excluded. Our data demonstrate that the process of p-body disassembly is controlled by phytochromes in R and FR light, with phyB playing a dominant role in R and phyA in FR light. Future work will have to unravel the mechanism by which the signal perceived by photoreceptors is transduced to a change in the composition and disassembly of p-bodies.

We used transgenic and mutant lines containing exclusively nuclear or cytoplasmic localised phyA to investigate which fraction of phyA is required and sufficient for the disassembly of p-bodies in response to FR light. As the cytoplasmic fraction of phyA is sufficient and the nuclear fraction does not elicit a disassembly of p-bodies, we conclude that the mechanism is not routed through nuclear phytochrome signalling. Until today, only few cytoplasmic phytochrome responses have been reported (Hughes, 2013). For example, it has been shown that phytochrome in the active Pfr state binds to PENTA 1 (PNT1) and thereby represses the translation of mRNAs, e.g., *PORA* mRNA (Paik et al., 2012).

It is still unclear if cytoplasmic phyB exerts a similar effect on p-bodies in response to R light as phyA does in response to FR light. Unfortunately, experimentally addressing this question for phyB is much more difficult than for phyA, because the nucleo-cytoplasmic partitioning of phyB is much less strict than for phyA (Klose et al., 2015b). Further studies could include crossing of p-body marker lines into a line expressing *p35S:PHYB-NLS-GFP* in *phyB-9* background to evaluate if nuclear phyB is able to trigger p-body disassembly. As no specific importer for phyB nuclear transport has been identified, the inverse, much more conclusive experiment is difficult. Here, fusions of phyB to GR could be of value to retain phyB in the cytosol and control nuclear import by application of DEX (Huq et al., 2003). Many responses mediated by phyA and phyB depend on the same signalling components (e.g., PIFs and COP1/SPA), so one can speculate that signal transduction downstream of these two photoreceptors also converges when it comes to regulation of p-body disassembly. Additionally, it will be of interest if the disassembly of p-bodies in response to R and FR light affects the translatome in a similar way, or if there are wavelength-specific effects. Jang et al. (2019) showed that mRNAs encoding OE33 and GUN5, proteins relevant for the establishment of chloroplasts, are released by p-body disassembly in light and undergo translation. As FR light plays a minor role in photosynthesis compared to R light, it will be interesting to evaluate if a different set of mRNAs is released from p-bodies in response to R and FR light.

In the dark, nuclear-localised COP1, together with SPA proteins, tags specific proteins for degradation by the proteasome. However, COP1 is also present in the cytosol (Balcerowicz et al., 2017) and therefore one could speculate that also cytosolic COP1 could be active and target a hypothetical protein X that degrades RNAs or proteins in p-bodies required for the structural integrity of p-bodies. When cytosolic COP1 is inhibited by photoactivated phytochromes in the cytosol, such a protein X would accumulate and could affect p-body composition and number (Supplementary Figure 8). To date, however, the identity of protein X is unknown and it is also unknown if COP1 has E3 ubiquitin ligase activity in the cytosol. Evidence for an involvement of COP1 in p-body disassembly is the fact that in a *cop1-6* mutant, the total p-body number is reduced and no further reduction takes place after light irradiation (Jang et al., 2019). Elucidating the mechanism of p-body disassembly will be crucial to understanding how light affects mRNA translation. Recent advancements in proteomic measurements of p-body contents purified from mammalian cell cultures might be useful to establish an experimental protocol to compare p-bodies isolated from light- and dark-grown plants (Hubstenberger et al., 2017).

As membraneless organelles, p-bodies are being formed by liquid-liquid-phase-separation (LLPS). The ability to undergo LLPS is depending on different factors, including an elevated local concentration of proteins that are of high intrinsic disorder. Additionally, *in vitro* studies showed that ribonucleoproteins (RNPs) bound to mRNAs can confer phase separation potential (Garcia-Jove Navarro et al., 2019). A process that disassembles p-bodies is presumably reducing this potential for phase separation by either selective or general removal of factors conferring phase separation potential. Identifying these factors will help understand how environmental cues, such as light, affect this process.

Even though NOT9B is a component of p-bodies implicated in light signalling and directly interacting with the FR photoreceptor phyA, there is no evidence that it is mechanistically involved in the assembly or disassembly of p-bodies. The phyA-binding capability of NOT9B is not relevant for the assembly of NOT9B into p-bodies, as mutating the PNB site that confers phyA binding capability of NOT9B does not lead to an altered pattern of p-body formation or disassembly. Since for the human homologue of NOT9B, CNOT9, a nucleotide-binding capacity was demonstrated *in vitro* (Garces et al., 2007), one could speculate that tethering of NOT9B to halted mRNAs localised to p-bodies might contribute to NOT9B’s dynamic association with p-bodies. Another potential mechanism for the association of NOT9B with p-bodies might be the interaction with GW-repeat proteins, a class of proteins that contain glycine/tryptophane repeats known to interact with AGOs and the human NOT9B homologue CNOT9 (Chen et al., 2014). A mutant version of NOT9B that lacks GW binding sites shows reduced association with p-bodies (Schwenk et al., 2021).

Structurally, CNOT9 consists of an Armadillo repeat (ARM) domain flanked by an N-terminal and a C-terminal unstructured, flexible region (Garces et al., 2007), a structure conserved in NOT9B. These regions of high intrinsic disorder could be considered as low-complexity domains, similar to low-complexity domains implicated in the formation of LLPS granules. The unstructured N-terminal domain of NOT9B was found to be phosphorylated in high-throughput studies (Heazlewood et al., 2008; Reiland et al., 2009). Phosphorylation might contribute to the nucleo-cytoplasmic partitioning, but also to the association with p-bodies under different light conditions. Experiments using different truncated and/or mutated versions of NOT9B will help elucidate whether it is a structural component of processing bodies.

With a saturation after approximately 4 h, the disassembly of p-bodies is a fast response. The initial release of mRNAs for translation is probably even faster, as we only observe the final disappearance of p-bodies. The fast kinetics is in line with the proposed purpose of releasing mRNAs quickly in order to adapt to a changing environment. Investigating the kinetics of p-body disassembly is therefore of considerable interest and FISH probes for identified p-body localised mRNAs or miRNAs could help understand the kinetics of their release.

It is tempting to speculate about why only approximately 50% of p-bodies disassemble after irradiation with light, irrespective of the duration and intensity of the light treatment. Literature describes a heterogeneous composition of p-bodies, which might contain different structural elements with different sensitivities to different stimuli, for example to hypoxia and light (Sorenson and Bailey-Serres, 2014; Jang et al., 2019). The term “processing body” presumably describes a heterogeneous class of different granules that have partially specific, partially overlapping contents and functions. This could explain how different sets of RNAs can be released in response to different stimuli and why only approximately 50% of p-bodies disassemble in response to light. Fluorescent Activated Particle Sorting (FAPS) could be used to purify p-bodies from plant tissue and separate them from the diffuse fraction. These purified bodies can be analysed for their protein and RNA content using MS/MS and RNA-seq techniques. Performing these experiments following treatments of plants with different stimuli that reduce the number of p-bodies could help distinguish between potential subclasses of p-bodies and identify their RNA and protein contents. Again, FISH probes could turn out to be an indispensable tool to investigate whether mRNA species are distributed equally throughout these different p-bodies. Not knowing the content and not knowing how the content is released from p-bodies is a current shortcoming in this fascinating field of research and one can expect great insight into the function of p-bodies once these questions are solved.

Taken together, our results reveal a novel cytoplasmic function of phyA in releasing mRNAs by disassembly of p-bodies in response to light. Our understanding of p-bodies is currently focussed on their RNA content, yet the molecular mechanism of the disassembly of p-bodies is still unclear. It will be interesting to find out whether light is the only stimulus that triggers the release of mRNA from p-bodies, or if this is a general mechanism in the regulation of plant development.

## Materials and Methods

### Plant Growth

For experimental purposes, plants were grown on 1/2 MS, 1.2% agar. Seeds were surface sterilised by incubating in 1 ml 70% EtOH for 10 min, followed by incubation in 1 ml 100% EtOH for 10 min. Seeds were left to dry in sterile conditions. After sowing, plates were kept for 48–72 h in 4°C for stratification. For germination induction, seeds were treated with 70 μmol m^–2^ s^–1^ white light for 6–8 h and transferred back to D for 4 days. Further light treatments were performed as described in the figure legends.

For breeding and propagation purposes, plants were grown on standard soil in a growth chamber in 100 μmol m^–2^ s^–1^ PAR in long-day conditions (16 h W, 22°C; 8 h D, 18°C).

### Light Treatments and Light Sources

For experiments, 740 nm LEDs have been used for FR light, 656 nm LEDs for R light, and fluorescent bulbs mounted in a Sanyo cabinet (Sanyo, Osaka, Japan) for W light. For plant cultivation, plants were kept under fluorescent light bulbs. Spectra of all light sources can be found in Supplementary Figure 9.

### Imaging and Counting of Processing Bodies

Seedlings were mounted in ddH_2_O in green light conditions and subjected to epifluorescence microscopy. Microscopic images were acquired using a Zeiss Axioplan 2 MOT (Carl Zeiss, Göttingen, Germany) equipped with a Photometrics CoolSNAP-HQ 12-bit monochrome CCD camera (Roper Scientific, Tucson, AZ, United States), external filter wheels (LUDL, Hawthorne, NY, United States), and filter sets for YFP (F31-028, excitation 500 nm, emission 515 nm; AHF Analysentechnik, Tübingen, Germany), or CFP (F31-044, excitation 436 nm, emission 455 nm; AHF Analysentechnik, Tübingen, Germany).

The upper third of the hypocotyl was used for imaging. Here, the uppermost layer of the epidermis was focused and three consecutive images along the hypocotyl were taken. A 350 × 200 px area was chosen of each picture in a way that as many p-bodies as possible were included. Using ImageJ, the number of p-bodies was counted.

### Protein Extraction and Western Blotting

Plants were grown for 4 days in D, followed by the indicated light treatments. 100 mg of plant material was harvested and ground in liquid nitrogen. 250 μl of 95°C SDS sample buffer [65 mM Tris/HCl pH 7.3, 4 M Urea, 3% SDS, 10% Glycerol, 0.05% Bromphenol blue, 20 mM DTT, 1× Protease Inhibitor Cocktail (Sigma-Aldrich, Cat-No: I3911)] was added and samples were incubated under vigorous shaking at 95°C for 10 min. Insoluble debris was pelleted by centrifugation (15 min, 20,000 × *g*). The supernatant was transferred to a new tube and protein content was determined using the Amido Black method (Popov et al., 1975).

Equal amounts of proteins were separated on a 10% SDS-PAGE and transferred to PVDF membrane. Membranes were blocked with 5% skim milk powder in PBS-T (137 mM NaCl, 2.7 mM KCl, 10 mM Na_2_HPO_3_, 1.8 mM KH_2_PO_4_, pH 7.4, 0.5% Tween-20). Equal loading was shown by Amido Black staining or detection of ACT. Membranes were probed with anti-HA-antibody (mouse, monoclonal, 1:1,000 in PBS-T, BioLegend, San Diego, CA, United States, Cat-No: 901533) or anti-ACT-antibody (mouse, monoclonal, 1:5,000 in PBS-T, Sigma-Aldrich, St. Louis, MO, United States, Cat-No: A0480) followed by secondary anti-mouse antibody (1:7,500 in PBS-T, Vector Laboratories, Burlingame, CA, United States, Cat-No: AP-2000-1). Immunodetection was performed using CDP-STAR (Sigma-Aldrich, Cat-No: 11759051001) according to manufacturers instructions. Quantification was done as described previously (Schwenk et al., 2021).

### Data Visualisation

Microscopy images were cropped and brightness/contrast adjusted using ImageJ. Plots were created using the Matplotlib package in Python 3.7.6 using Spyder IDE v4.0.1. Box plots display the following features of the data sets: White line indicates the median value, and box indicates the limits of quartile 1 (Q1) and quartile 3 (Q3). Interquartile range (IQR) is defined as Q3 – Q1. Whiskers indicate Q1–1.5 × IQR and Q3 + 1.5 × IQR. Circles indicate outliers that do not fall into the whisker range.

Figures were assembled using Inkscape. Statistical analysis was performed as indicated in the figure legends.

### Plasmid Constructs and Plant Lines Used in This Study

All plasmid constructs and plant lines used in this study are listed in Supplementary Tables 1, 2 (Genoud et al., 2008; Rausenberger et al., 2011; Menon et al., 2020; Schwenk et al., 2021).

## Data Availability Statement

The original contributions presented in the study are included in the article/Supplementary Material, further inquiries can be directed to the corresponding author/s.

## Author Contributions

PS and AH: conceptualisation, visualisation, writing – original draft, writing – review and editing, and funding acquisition. PS: investigation. AH: project administration. Both authors contributed to the article and approved the submitted version.

## Conflict of Interest

The authors declare that the research was conducted in the absence of any commercial or financial relationships that could be construed as a potential conflict of interest.

## Publisher’s Note

All claims expressed in this article are solely those of the authors and do not necessarily represent those of their affiliated organizations, or those of the publisher, the editors and the reviewers. Any product that may be evaluated in this article, or claim that may be made by its manufacturer, is not guaranteed or endorsed by the publisher.

## Supplementary Material

Supplementary Table 1 List of plasmid constructs used in this study.

Supplementary Table 2 List of *A. thaliana* plant lines used in this study.
