# Supplementary material for: Phytochrome A Mediates the Disassembly of Processing Bodies in Far-Red Light
Source: Front Plant Sci. 2022 Feb 23;13:828529. doi: 10.3389/fpls.2022.828529 (PMC8905148; doi:10.3389/fpls.2022.828529)
Supplement: Supplementary Table 1 — List of plasmid constructs used in this study. [file Table_1.pdf]

Suppl. Tab. 1: List of plasmid constructs used in this study.

Plasmid construct	Reference
p35S:HA-YFP-NOT9B	(Schwenk et al., 2021)
p35S:DCP1-CFP	(Schwenk et al., 2021)
pPHYA:PHYA-CFP	(Genoud et al., 2008)
pPHYA:PHYA-NLS-YFP	(Rausenberger et al., 2011)
p35S:HA-YFP-NOT9B ΔPNB	(Schwenk et al., 2021)

References

Genoud, T., Schweizer, F., Tscheuschler, A., Debrieux, D., Casal, J. J., Schäfer, E., et al. (2008). FHY1 mediates nuclear import of the light-activated phytochrome A photoreceptor. *PLoS Genet.* 4, e1000143. doi:10.1371/journal.pgen.1000143.

Rausenberger, J., Tscheuschler, A., Nordmeier, W., Wüst, F., Timmer, J., Schäfer, E., et al. (2011). Photoconversion and nuclear trafficking cycles determine phytochrome A's response profile to far-red light. *Cell* 146, 813–825. doi:10.1016/j.cell.2011.07.023.

Schwenk, P., Sheerin, D. J., Ponnu, J., Staudt, A.-M., Lesch, K. L., Lichtenberg, E., et al. (2021). Uncovering a novel function of the CCR4-NOT complex in phytochrome A-mediated light signalling in plants. *eLife* 10, e63697. doi:10.7554/eLife.63697.
