# Supplementary material for: Phytochrome A Mediates the Disassembly of Processing Bodies in Far-Red Light
Source: Front Plant Sci. 2022 Feb 23;13:828529. doi: 10.3389/fpls.2022.828529 (PMC8905148; doi:10.3389/fpls.2022.828529)
Supplement: Supplementary Table 2 — List of A. thaliana plant lines used in this study. [file Table_2.pdf]

Suppl. Tab. 2: List of *A. thaliana* plant lines used in this study.

**Plant lines**					
**Transgene or mutant**	**Genetic background**	**Line number**	**Generated via**	**Vector for floral dip**	**Reference/ internal seed stock identifier**
p35S:HA-YFP-NOT9B	Col-0	21c	Floral dip	DS361	(Schwenk et al., 2021) / #PS1704
p35S:YFP-HA	Col-0		Floral dip	#2969 pPPO30v1HA	(Schwenk et al., 2021)
phyA-211	Col-0				NASC 6223/ #PS1707
p35S:HA-YFP-NOT9B	*phyA-211*		Crossing of #PS1704 and #PS1707		(Schwenk et al., 2021) / #PS856
pPHYA:PHYA-CFP	*phyA-211*		Floral dip	#1361 (Genoud et al., 2008)	#8749
pPHYA:PHYA-NLS-YFP	*phyA-211*		Floral dip	#2095	#7129 (Menon et al., 2020)
p35S:HA-YFP-NOT9B	*pPHYA:PHYA-NLS-YFP*		Crossing of #PS1704 and #7129		(Schwenk et al., 2021) / #PS1831
p35S:HA-YFP-NOT9B	*pPHYA:PHYA-CFP phyA-211*		Crossing of #PS1704 and #8749		this study/ #PS526
p35S:DCP1-CFP	Col-0	4a	Floral dip	pPS74	this study/ #PS2051
phyB-9	Col-0				NASC N6217/ #PS1706
p35S:HA-YFP-NOT9B	*phyB-9*		Crossing of #PS1704 and #PS1706		(Schwenk et al., 2021) / #PS983
p35S:HA-YFP-NOT9B (H58A F60A A64Y V71Y) = p35S:HA-YFP-NOT9B ΔPNB	Col-0	7c	Floral dip	pMBag06	(Schwenk et al., 2021) / #PS1444

p35S:HA-YFP-NOT9B	*fhy1-3 fhl-1*		Crossing of #PS1704 and #2667/ *fhy1-3 fhl-1*		this study/ #PS1773
p35S:HA-YFP-NOT9B	*hy5-215*		Crossing of #PS1704 and *hy5-215*		this study/ #PS1857
fhy1-3 fhl-1	Col-0				#2667/ *fhy1-3 fhl-1*
Col-0	n/a				NASC N1092/ #4896

References

Genoud, T., Schweizer, F., Tscheuschler, A., Debrieux, D., Casal, J. J., Schäfer, E., et al. (2008). FHY1 mediates nuclear import of the light-activated phytochrome A photoreceptor. *PLoS Genet.* 4, e1000143. doi:10.1371/journal.pgen.1000143.

Menon, C., Klose, C., and Hiltbrunner, A. (2020). Arabidopsis FHY1 and FHY1-LIKE are not required for phytochrome A signal transduction in the nucleus. *Plant Comm.* 1, 100007. doi:10.1016/j.xplc.2019.100007.

Schwenk, P., Sheerin, D. J., Ponnu, J., Staudt, A.-M., Lesch, K. L., Lichtenberg, E., et al. (2021). Uncovering a novel function of the CCR4-NOT complex in phytochrome A-mediated light signalling in plants. *eLife* 10, e63697. doi:10.7554/eLife.63697.
