# Supplementary figures and images for: Phytochrome A Mediates the Disassembly of Processing Bodies in Far-Red Light
Source: Front Plant Sci. 2022 Feb 23;13:828529. doi: 10.3389/fpls.2022.828529 (PMC8905148; doi:10.3389/fpls.2022.828529)

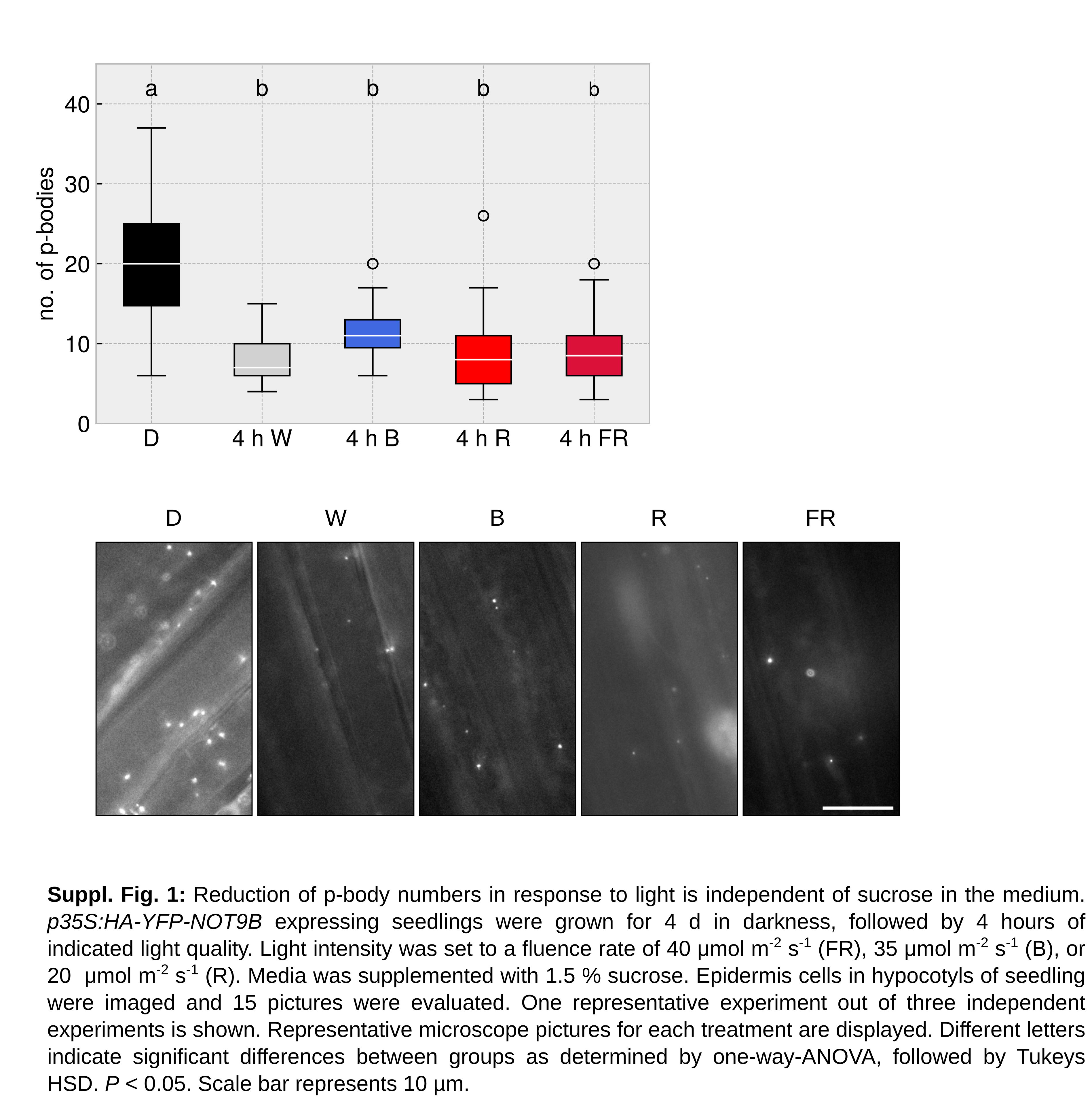

Supplement: Supplementary file 3 [file Image_1.JPEG]

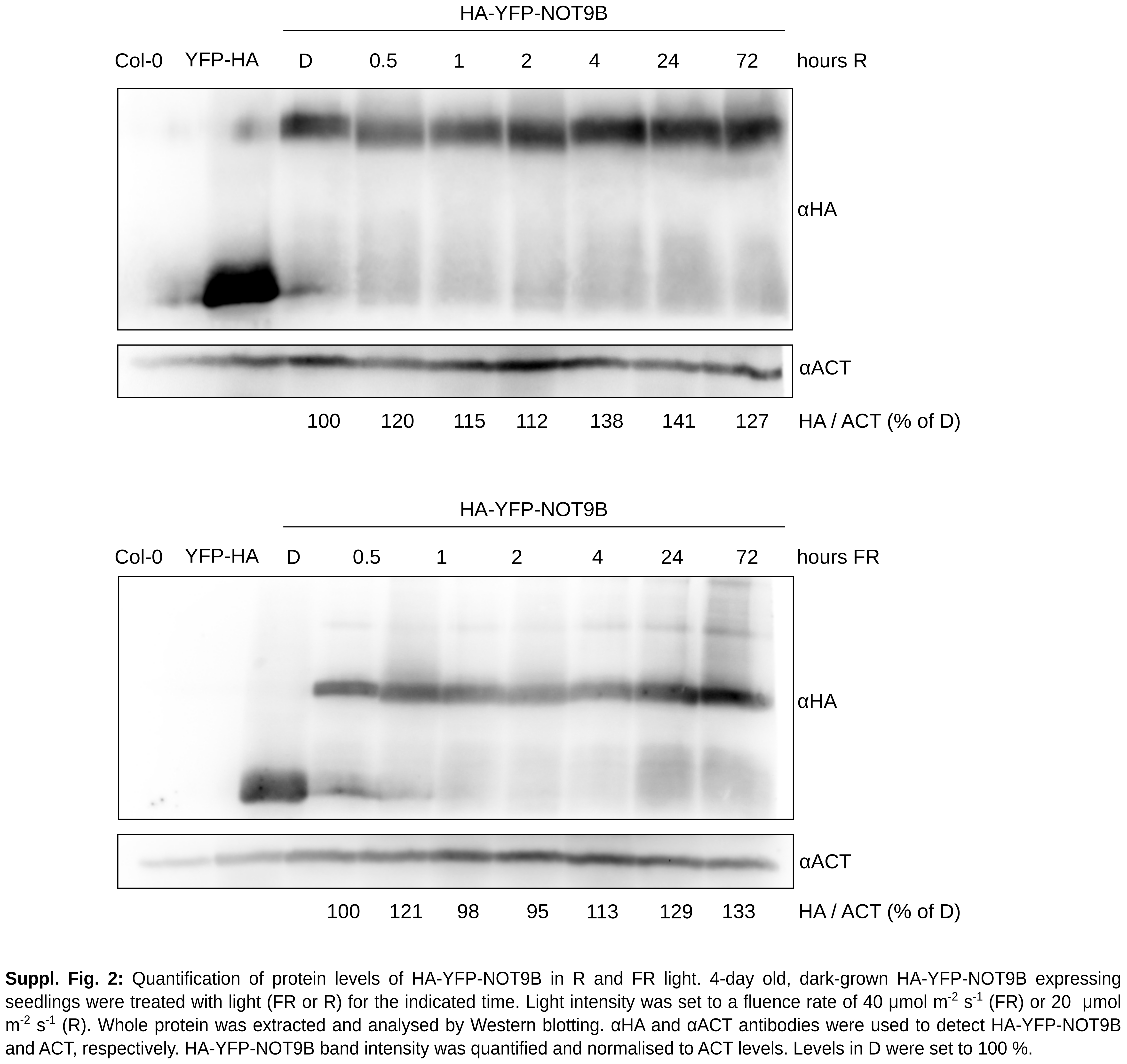

Supplement: Supplementary file 4 [file Image_2.JPEG]

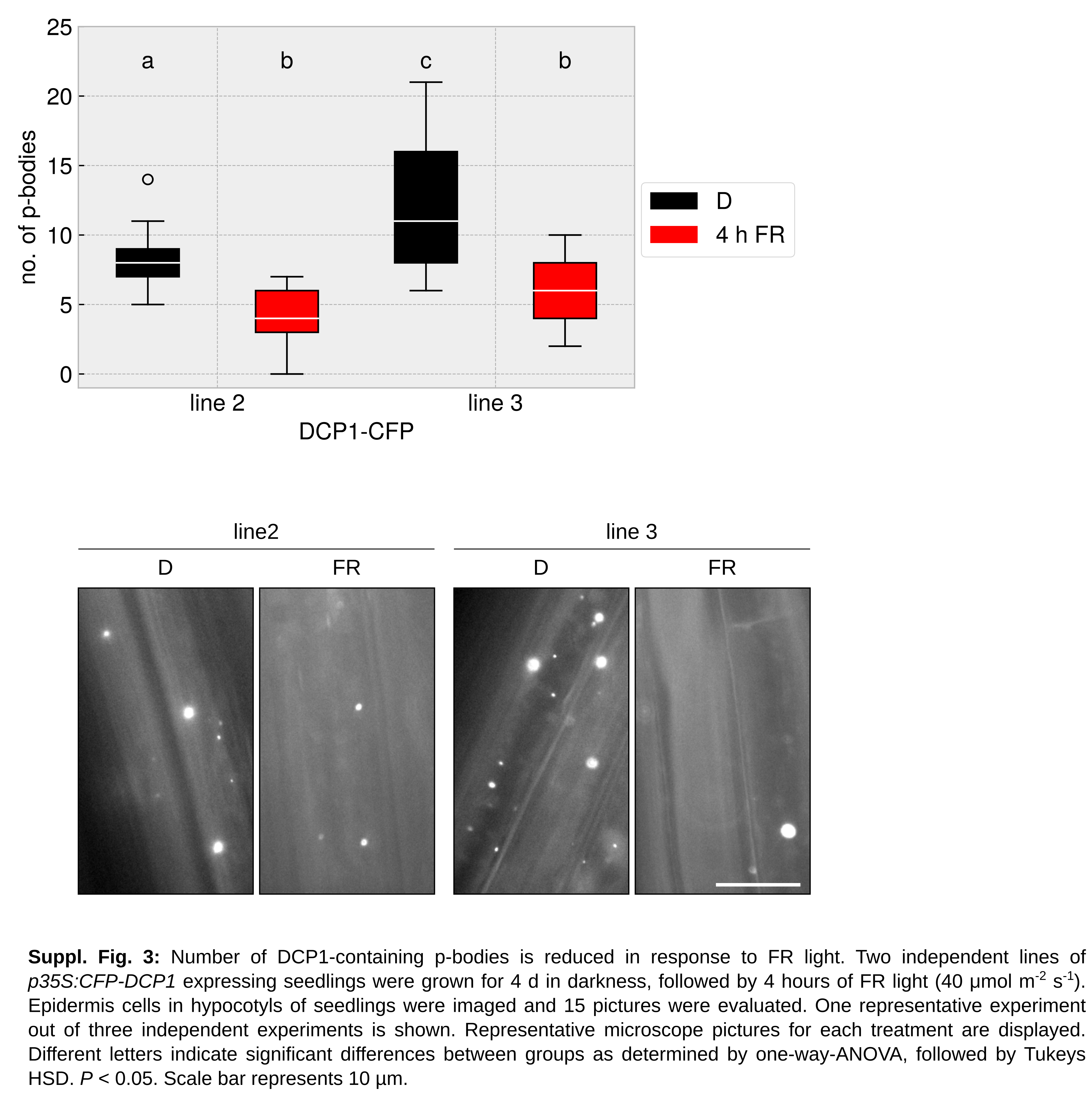

Supplement: Supplementary file 5 [file Image_3.jpg]

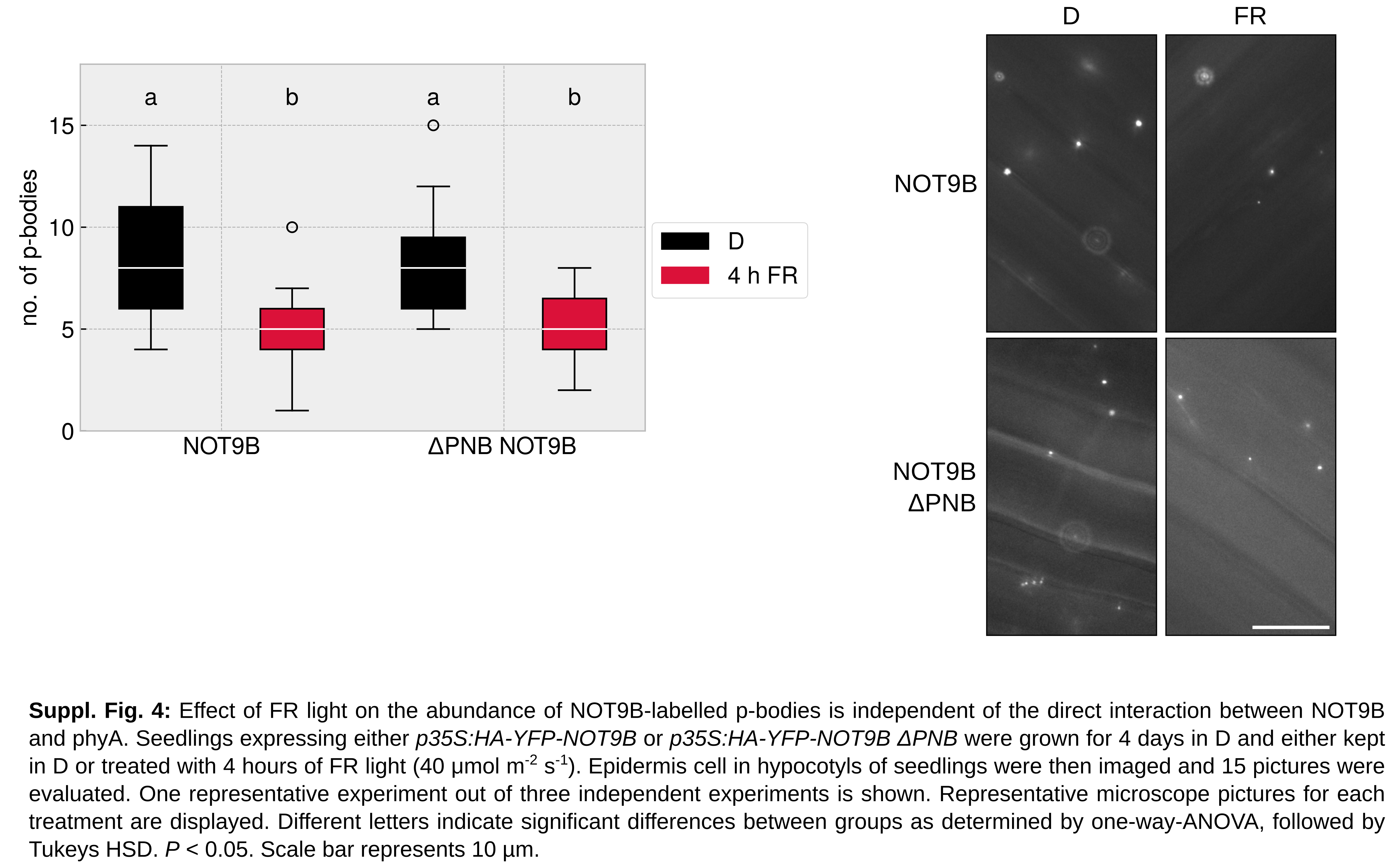

Supplement: Supplementary file 6 [file Image_4.jpg]

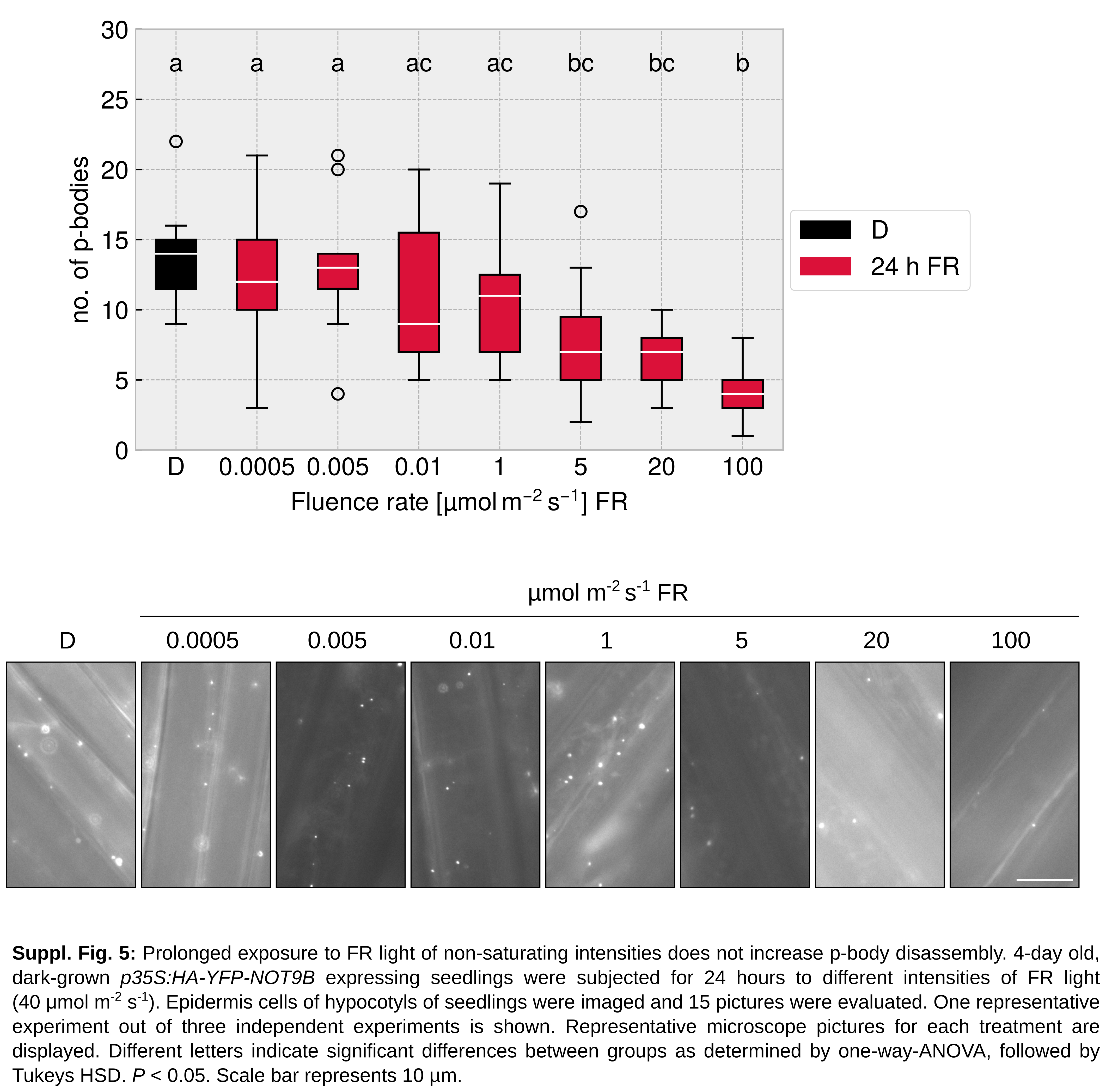

Supplement: Supplementary file 7 [file Image_5.JPEG]

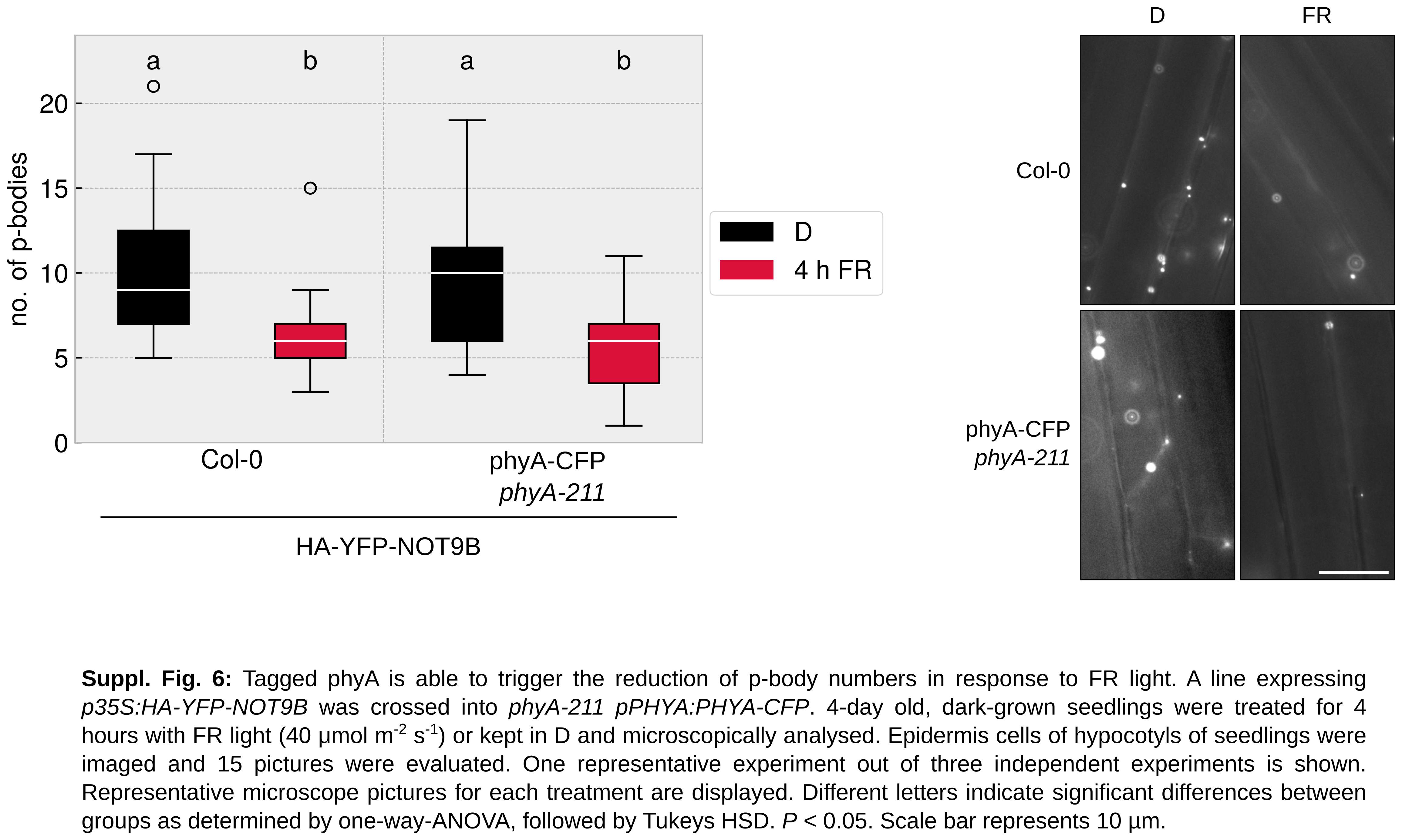

Supplement: Supplementary file 8 [file Image_6.JPEG]

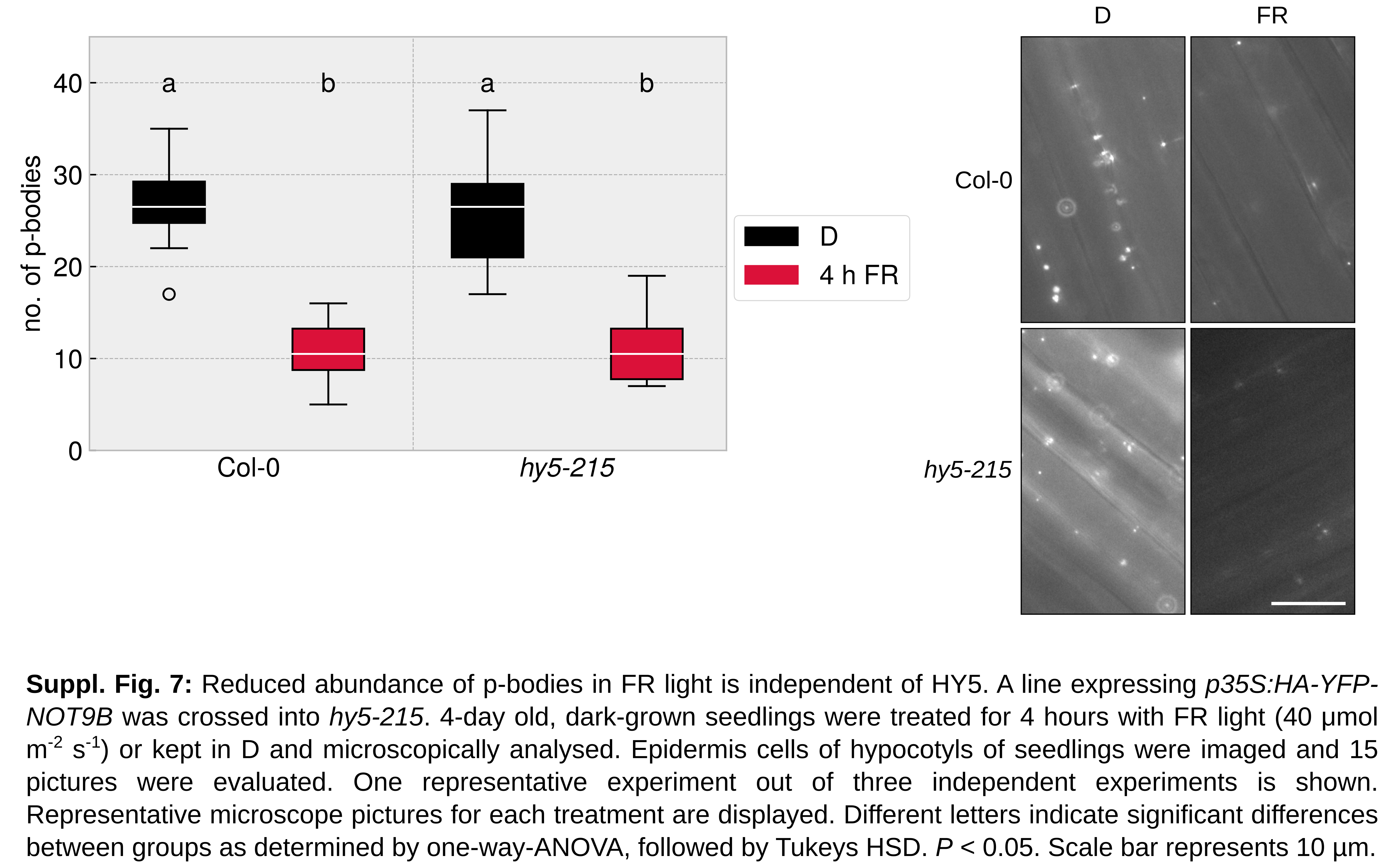

Supplement: Supplementary file 9 [file Image_7.JPEG]

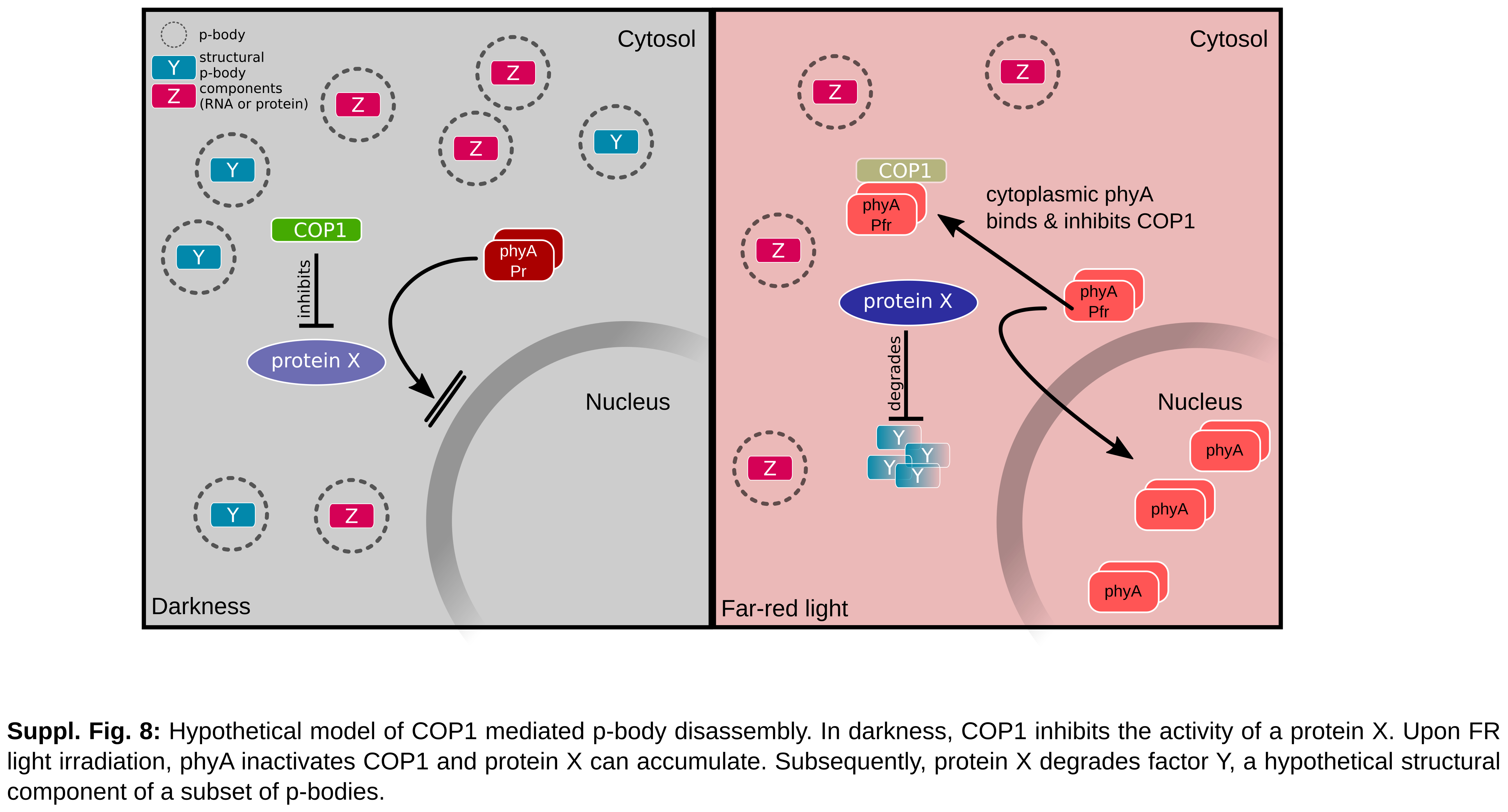

Supplement: Supplementary file 10 [file Image_8.jpg]

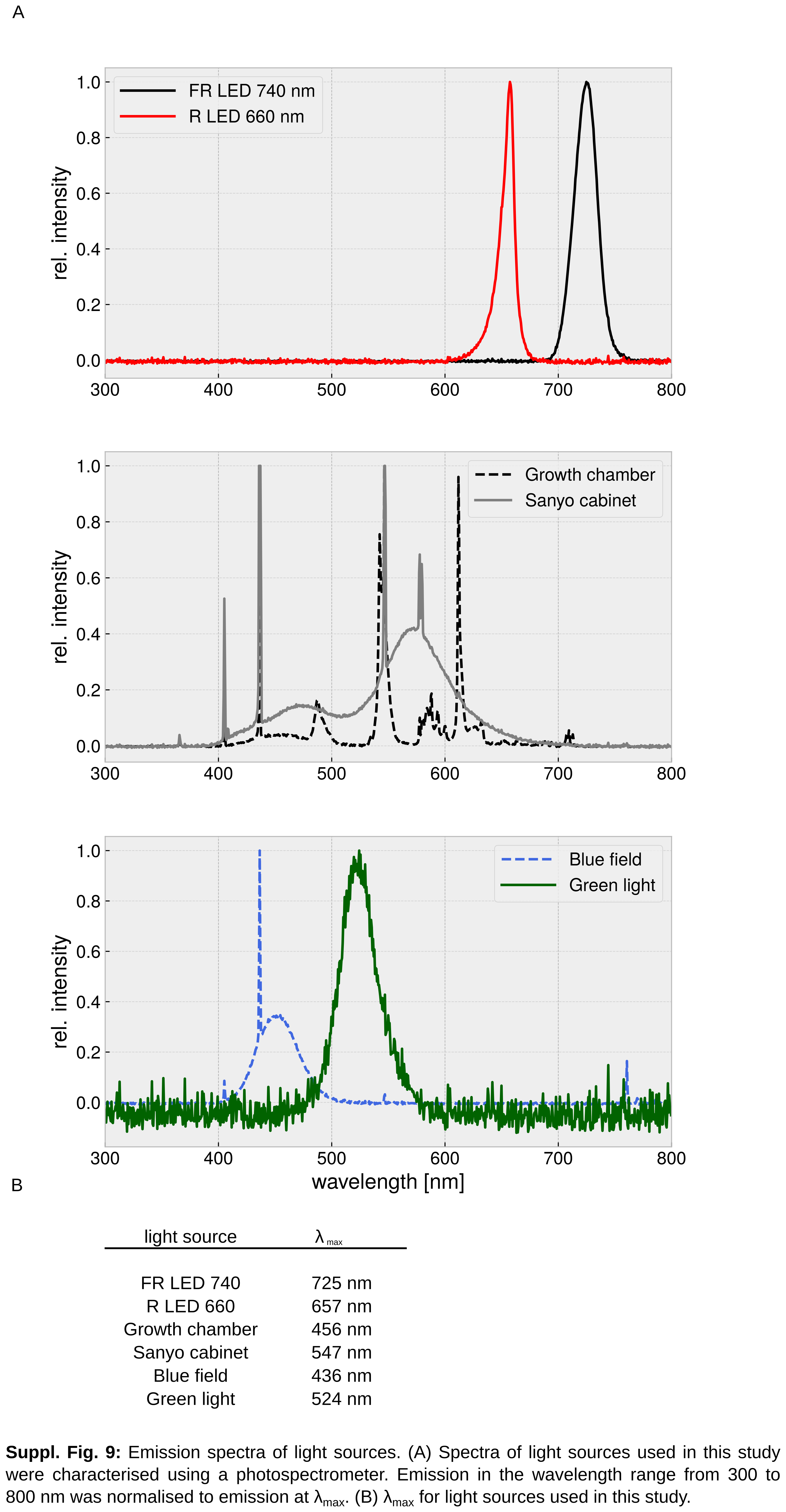

Supplement: Supplementary file 11 [file Image_9.JPEG]
